# Health Risk, Functional Markers and Cognitive Status in Institutionalized Older Adults: A Longitudinal Study

**DOI:** 10.3390/ijerph17197303

**Published:** 2020-10-07

**Authors:** Raquel Pedrero-Chamizo, Ulrike Albers, Gonzalo Palacios, Klaus Pietrzik, Agustín Meléndez, Marcela González-Gross

**Affiliations:** 1 ImFINE Research Group, Department of Health and Human Performance, Faculty of Physical Activity and Sport Sciences-INEF, Universidad Politécnica de Madrid, 28040 Madrid, Spain; ulrikealbers@gmail.com (U.A.); gonzalopalacios88@gmail.com (G.P.); amelendez8@msn.com (A.M.); marcela.gonzalez.gross@upm.es (M.G.-G.); 2 Exercise and Health in Special Population Spanish Research Net (EXERNET), 50009 Zaragoza, Spain; 3 CIBEROBN (Physiopathology of Obesity and Nutrition CB12/03/30038), 28029 Madrid, Spain; 4 Department of Nutrition and Food Science, Rheinische Friedrich-Wilhelms University, 53115 Bonn, Germany; k.pietrzik@uni-bonn.de

**Keywords:** cardiovascular biomarkers, cognitive function, functional capacity, muscle strength, older adults, geriatrics

## Abstract

A Follow-up of vitamin B12 and lipids status is essential in older people, being closely related to non-communicable diseases. Their relationships with cognitive and physical status are not clear. The aim was to analyze the evolution of vitamin B12 and related parameters, lipid and hematological profiles, and their relationships with cognitive and physical status among institutionalized elderly. Sixty residents, ranged from 62 to 99, were evaluated. Biomarkers (vitamin B12 and related parameters, lipid and hematological profiles), functional capacity (handgrip, arm and leg strength), and cognitive status (Mini-Mental State Examination) were evaluated four times at 4-month intervals. At the beginning of the study, 63% and 70% of the sample showed abnormal homocysteine and folate values, respectively. At the end of the year, abnormal homocysteine increased to 68%, abnormal folate values decreased to 50%. Throughout the year, serum folate showed a significant increase (14.9 vs. 16.3 nmol/L), (*p* < 0.05). Serum cobalamin (299 vs. 273 pmol/L). HDL-cholesterol (49.9 vs. 47.0 mg/dL) and triglyceride levels (102.4 vs. 123.2 mg/dL) showed a significant decrease and increase respectively in mean values (all *p* < 0.05). Serum cobalamin and HDL-cholesterol were the most important biomarkers associated with cognitive function (both *p* < 0.05). The most relevant biomarkers associated with poor physical strength depending on the body part analyzed were low concentrations of HDL-cholesterol, LDL-cholesterol, apolipoprotein A1, and albumin (all *p* < 0.05). The evolution of lipid biomarkers, their significance with cognitive values, and association with handgrip, point to the importance of the handgrip measurement, a very simple test, as an important health marker. Both serum albumin and physical strength are important health markers in older people.

## 1. Introduction

According to a new United Nations report, the world population is expected to increase by 2 billion persons, from 7.7 billion to 9.7 billion in the next 30 years [1]. The percentage of people aged (60 years or older) with respect to the total population increased from 9% in 1994 to 12% in 2014, and in 2050, it will probably reach 21% [2].

Aging is a multifactorial process, influenced by the passage of time and by functional, physiological, biological, and social changes [3]. As age increases, limitations due to health conditions become more apparent causing increasing disability and, consequently, a reduction in self-perceived quality of life. Disability is present in less than 1 in 10 people up to 44 years, compared to more than 5 in 10 among those over 74 years but it is in the group of 85 years and more where disability is more widespread [4].

Gerontology is broadening its perspective from a prior preoccupation with disease and disability to a more comprehensive view that includes successful aging, and maintaining and improving the quality of life are some of the main challenges posed by the aging process [4,5,6]. Successful aging is defined as including three main components: low probability of disease and disease-related disability, high cognitive and functional capacity, and active engagement with life. Low probability of disease refers not only to absence or presence of disease itself but also to the absence, presence, or severity of its risk factors. High functional levels include both physical and cognitive components, which tell us what a person can do [5].

Two families of biomarkers are usually used to monitor the health of older people assessing cardiovascular risk factors, those related to lipids (total-cholesterol, HDL-cholesterol, LDL-cholesterol, triglycerides, apolipoprotein A1, apolipoprotein B) [7], and those related to vitamin B12 status (serum cobalamin, holo-transcobalamin, serum and red blood cell folate, and homocysteine) [8]. However, studies reporting results simultaneously from both types of biomarkers, muscular strength and cognitive status, and their evolution are scarce, especially for seniors living in homes for older adults. The purposes of this study were to monitor over the time and investigate the relationship among these parameters over one year, in institutionalized older adults.

## 2. Materials and Methods

### 2.1. Study Population

The study was performed in the framework of a broader project dealing with the early diagnosis and treatment of vitamin B12 deficiency in Spanish older adults of both sexes [9]. Participants were recruited from three nursing homes in the Madrid Region (Spain). The study was performed with the participation of the responsible physician from each residence. The study design was prospective longitudinal with a one-year follow-up. Inclusion criteria were to be institutionalized older adults aged 60 or above.

Exclusion criteria were cobalamin or folate supplement intake, neuropathy, diagnosed severe dementia, Hunter’s glossitis, and any disease that might prevent normal execution of most of the physical tests. Data on medication intake (for chronic as well as acute diseases) was collected from the data management systems of the nursing homes [10]. The concomitant intake of medication known to affect cobalamin absorption (such as metformin, H2-antagonists, and proton pump inhibitors) was not an exclusion criterion if the medication had been started at least three months before the study and was taken continuously by the study participant for its whole duration. Furthermore, splitting the whole study population into two groups (taking or not taking medication with a possible influence on vitamin B12 status) did not result in statistical differences, according to a previously analysis made on this population sample [10]. The study was conducted in accordance with the ethical principles of the Helsinki Declaration of 1964 and later amendments, with the Convention on Human Rights and Biomedicine of Oviedo in 1997 and approved by the Human Research Review Committee of the University of Granada. A written informed consent was obtained from all study participants and/or family representatives or guardians.

### 2.2. Blood Sampling

Blood was collected from the participants after an overnight fast, four times over a total of one year (at month 1, 5, 9, and 13). Blood specimens were collected in Vacutainer tubes containing EDTA or gel for serum. The EDTA tubes were used for whole blood count and red blood cell folate (RBC folate), and the gel tubes were immediately placed on ice. After blood clotting, they were centrifuged at 3000 g, aliquoted for serum samples and frozen at −86 °C until analysis.

### 2.3. Biomarker Measurements

Serum cobalamin (sCbl) concentrations were measured with a Microparticle Enzyme Immunoassay (MEIA, Abbott Diagnostics, Abbott Park, IL, USA, total CV < 11%) [11], holotranscobalamin (holoTC) by a two-step sandwich MEIA (Abbott AxSYM, Abbott Park, IL, US, total CV < 10%) [12], serum folate and RBC folate with an ion-capture immunoassay (ICIA; Abbott AxSYM, total CV < 19%) [13], serum total homocysteine (tHcy) with a fluorescence polarization immunoassay (FPIA; Abbott AxSYM, Abbott Park, IL, US, total CV < 6%) [14]. Analyses were performed in the biochemical laboratory of the Faculty of Sport Sciences, UPM, Madrid (Registered Lab number 242, Red de Laboratorios de la Comunidad de Madrid, Madrid, Spain).

Total-cholesterol, HDL-cholesterol, LDL-cholesterol, triglycerides, apolipoprotein A1 (ApoA), apolipoprotein B (ApoB), lipoprotein A (LpA), glucose, albumin, and creatinine were obtained by standard spectrophotometric assays on a Hitachi 912 (Roche Diagnostics, Mannheim, Germany) conducted by a local certified clinical chemistry laboratory (CLIMESA, Gabinete Médico Conde-Duque, Madrid, Spain). For both biomarker families, cut-offs for abnormal values were those used routinely in each laboratory and are indicated in the footnotes of Table included in Supplementary Material Table S1.

### 2.4. Functional Capacity

Three different muscle strength tests were used for the assessment of functional capacity. Maximal handgrip strength (HGS) was measured using a dynamometer Takei TKK 5101 (Takei Scientific Instruments Co Ltd, Niigata, Japan), range 5–100 Kg with a precision 0.1 Kg. Participants sat with arm extended and parallel to the body and without moving the wrist [15]. Two trials were recorded on both hands with at least 1-min rest between them. The average value was included in the statistical analysis.

Upper body strength was assessed with the arm curl test [16] whose aim is to do as many arm curls as possible in 30 s holding a 2 kg (women) or 4 kg (men) dumbbell in the hand of the dominant arm [15]. Participants performed two repetitions (5-min apart), and the mean of these trials was used for analysis.

Lower body strength was evaluated by the 30-s chair-stand test [16]. Briefly, participants sat on a chair and were asked to rise to a full stand and to sit down again. They repeated this cycle for 30 s so that the test score corresponds to the number of cycles performed during this time. The test was performed only once.

### 2.5. Cognitive Status

The Mini-mental state examination (MMSE) [17] is the most widely used standardized tool for measuring cognitive impairment in clinical practice and research. The original version translated to Spanish was used (Psychological Assessment Resources (PAR), Inc., Lutz, FL, USA). It is composed of an 11-question measure that tests for functions including arithmetic, memory, and orientation. Total score ranges from 0 to 30, with lower values indicating greater impairment. Cognitive impairment cut-off for MMSE test was set to be below 24 [18].

### 2.6. Confounders

Potential confounders were selected from the literature and included demographics (gender, age and educational level) and body mass index (BMI; kg/m^2^). All analyses presented are adjusted for these covariates.

### 2.7. Statistical Analysis

To study the evolution of the means along a year, a mixed statistical model was applied. Dependent variables were MMSE and functional scores with time as the repeated and random effect.

In a first approach to determine which biomarkers showed an association with these dependent variables, a Pearson’s linear correlation was performed. Biomarkers that showed an individual correlation with cognitive and physical functions (muscular strength) were selected; an additional linear mixed model test was carried out, in order to eliminate confounding effects between biomarkers, by considering these functions as dependent variables, and biomarkers as covariates. Time was assessed as not having effect on either dependent variables or covariates.

In all cases, continuous variables were checked for Gaussian normal distribution with the Kolmogorov–Smirnov test, and whenever a normal distribution could not be achieved with raw data, logarithmic transformation was applied. Statistics SPSS 21.0 software (IBM, Chicago, IL, USA) was used to analyze data from subjects with complete biochemical, cognitive, and physical data. Statistical significance was set at *p* < 0.05.

## 3. Results

**Figure 1:**
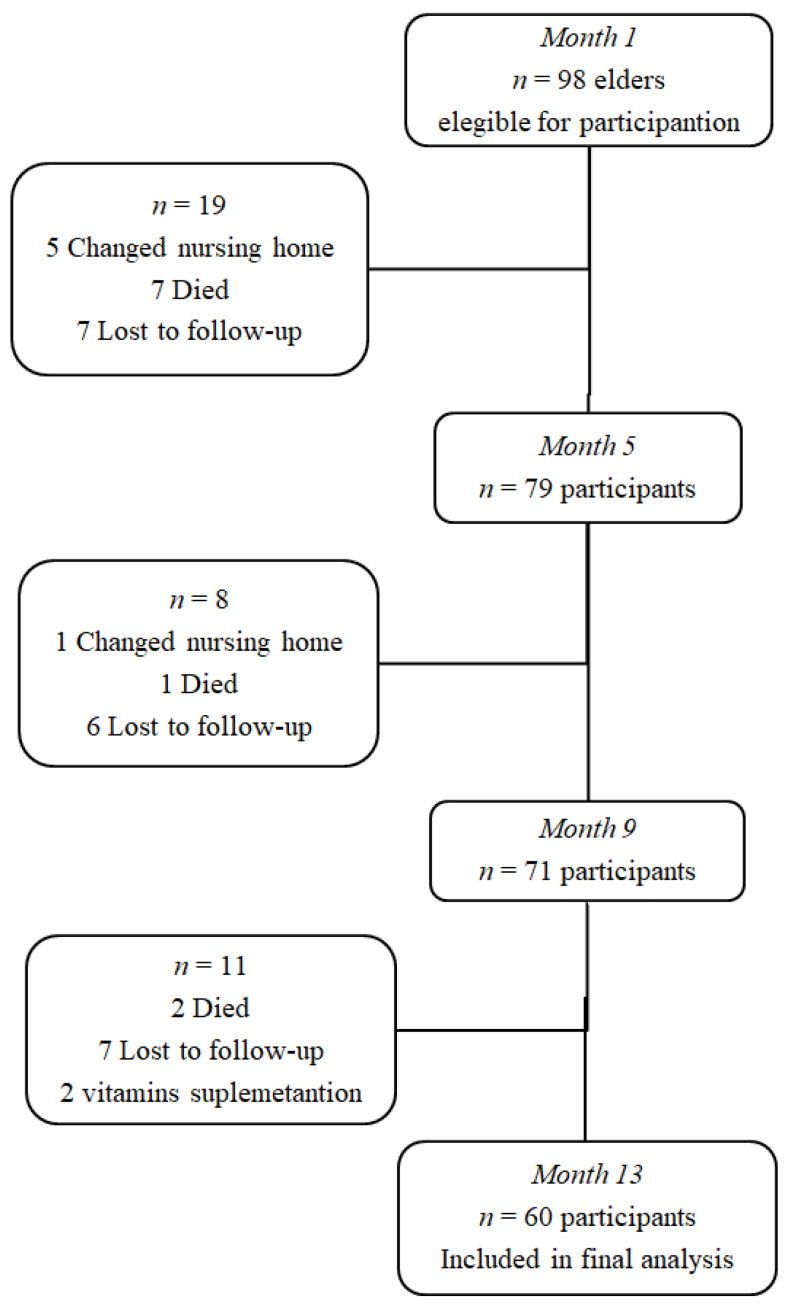
Flow chart of participants in the study.

The sample size was calculated on the basis of the most highly fluctuant variable (HoloTC) among the main outcomes and the statistical tests used (dependent samples with four examination points). Based on this, the necessary sample size was 60 subjects. In order to achieve the study sample population size, 98 participants were initially recruited. During the follow-up period, 36 participants (39%) were lost because they moved out of the study area or died, and another two were excluded because they started folate/cobalamin supplementation during the observation period so that; finally, the data of 60 participants (41 women-19 men) were included for analysis (see, Figure 1). Table 1 shows the baseline (month 1) characteristics of this population.

### 3.1. Evolution of Biomarkers and Cognitive and Physical Tests over a Year

The evolution of biomarkers and functional and cognitive tests, as well as the prevalence of abnormal values over one year, are summarized in Supplementary Table S1.

The prevalence of the tHcy value above normal values was high at month 1 (63%) and continued to be elevated at the end of the study at month 13 (68%). Serum folate (70%) and HoloTC (32%) deficiencies were present at the beginning of the study, and a low decrease was observed a year later (50% and 25%, respectively). Prevalence of sCbl and RBC folate values deficiencies were 10% and 3%, respectively, at the beginning, but RBC folate increased to 12% at the end of the study.

**Figure 2:**
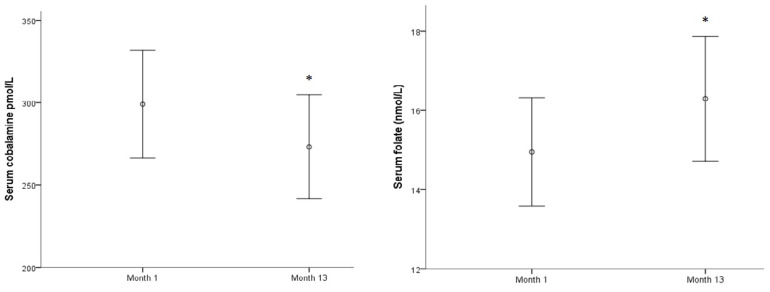
Serum cobalamine and serum folate evolution along a year.

In spite of these prevalence variations, no significant changes were observed in the means of each biomarker along this period of time, except for sCbl, in which the mean decreased, and for serum folate, in which the mean increased slightly (both *p* < 0.05) (Figure 2).

**Figure 3:**
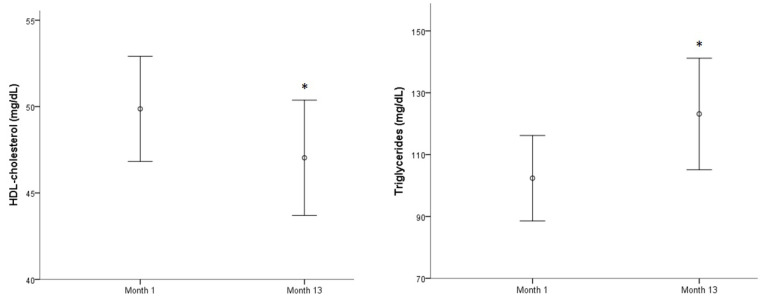
HDL-cholesterol and triglycerides evolution along a year.

Two biomarkers related to lipid profile worsened significantly throughout the year: Mean HDL-cholesterol decreased (*p* < 0.05) while mean triglycerides showed an important increase (*p* < 0.001) (Figure 3). The other means showed no significant differences throughout the year. The highest prevalence biomarker concentrations above reference range were observed first for LpA (52–53%) followed by LDL-cholesterol (33–38%) and total-cholesterol (30–38%). The number of subjects presenting low HDL-cholesterol values increased over a year (13–22%) while high triglyceride concentrations were found in 10% of the population at the beginning of the study and in 20% of subjects at month 13.

Neither creatinine nor albumin or glucose showed significant differences throughout the study. Prevalence of glucose above normal range was high (around 45%) and quite irregular, over the follow-up period. The mean of physical test results and MMSE scores did not differ significantly over the time-study period.

### 3.2. Association between Biomarkers and MMSE Score

When assessing bivariate correlation of each biomarker with cognitive status (Table 2), MMSE scores showed a significant positive correlation with sCbl, HDL-cholesterol, ApoA, and albumin. A significant negative correlation was obtained for LDL-cholesterol and triglycerides, and a clear negative tendency with total-cholesterol (*p* = 0.051). If biomarkers were incorporated simultaneously as covariates (Table 3), only sCbl and HDL-cholesterol remained associated with cognitive status.

**Figure 4:**
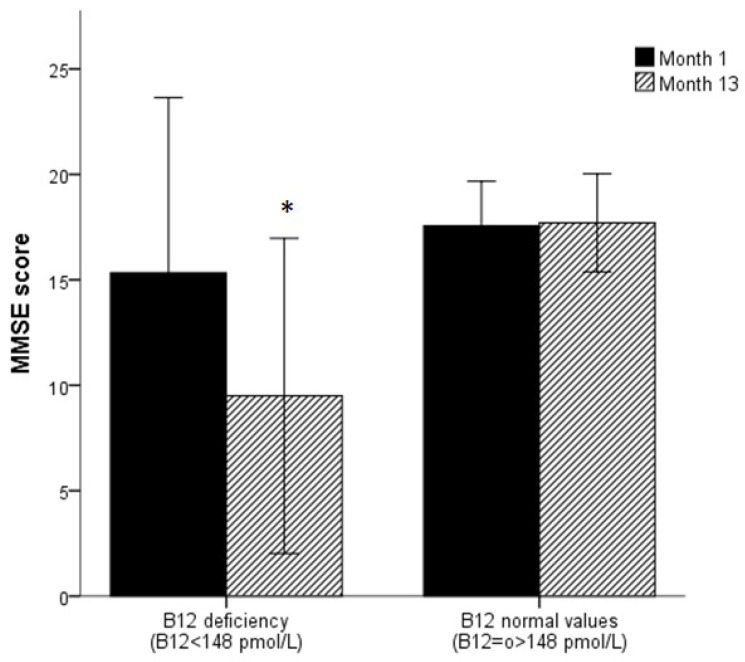
MMSE score evolution along a year according to abnormal vitamin B12 cut-off.

It is important to note that, subjects with sCbl deficiency at the beginning of the study showed lower MMSE scores (*p* < 0.001) and there was a worsening of the MMSE scores for the sCbl deficiency group one year later. Subjects with normal sCbl values showed similar MMSE scores at the beginning and the end of the study (Figure 4)).

### 3.3. Association between Biomarkers and Functional Tests

When biomarkers were studied separately for correlation assessment, HGS only showed a significant positive correlation with albumin. Significant negative correlations with HGS were observed for RBC folate, total-cholesterol, LDL-cholesterol, and triglycerides (Table 2). Biomarkers, except HDL-cholesterol lost their association with HGS when observed as covariates. HDL-cholesterol became the sole marker presenting a positive significant association (Table 3).

The biomarkers showing a positive significant correlation with leg strength were ApoA, ApoB, and albumin. When studying biomarkers as covariates, LDL-cholesterol and albumin showed a positive association with leg strength.

Finally, albumin, ApoA, and HDL-cholesterol showed individually a positive correlation with arm strength and a negative association with LDL-cholesterol (Table 3). When studying these variables simultaneously, ApoA and albumin remained significantly and positively associated with arm strength (Table 3).

## 4. Discussion

The importance of the present report is to study for the first time (to the best of our knowledge), the association of commonly available biomarkers related to vitamin B12 and lipid metabolism with physical strength (hand, arm and leg individually studied) and cognitive status, and their evolution in an older adult institutionalized population over one year. Significant associations found in this study between blood markers and cognitive and functional testing were slight.

### 4.1. Evolution of Biomarkers and Cognitive and Functional Tests over a Year

The most important variations observed in this population throughout a year were that sCbl and HDL-cholesterol decrease and triglycerides increase.

In general, for B12-related parameters, significant differences were only observed for sCbl and serum folate, without any difference in the percentage of cases with abnormal values. However, prevalence of tHcy above the normal values changed from 63% at month 1 to 68% at month 13.

Serum folate and HoloTC deficiencies at month 1 (70% and 32%) decreased unexpectedly (50% and 25%, respectively), which makes us wonder if there was a possible uncontrolled supplementation during the observation period. RBC folate deficiency (3%) was low at the beginning and increased to 12% at the end of the study.

Two biomarkers of cardiovascular risk related to lipid profile worsened significantly throughout the year: Mean HDL-cholesterol decreased while mean triglycerides showed an important increase. Participants presenting low HDL-cholesterol increased from 13% to 22%, and those experienced an increase in triglyceride levels increased from 10% to 20%.

Neither creatinine nor albumin or glucose showed significant differences throughout the study. Prevalence of glucose above normal range was high (around 45%) and quite irregular, over the follow-up period. Mean of physical test results and MMSE scores did not differ significantly over the time-study period.

### 4.2. Biomarkers and Cognitive Status

In our study, the only biomarker related to vitamin B12 metabolism and associated with cognitive status, assessed by MMSE test, was sCbl. These results were consistent with the findings from some previous longitudinal studies, all performed in free-living older people, like in the Rotterdam Study with a follow-up of 2.7 years [19], the Leiden 85-Plus Study (4 years follow-up) [20], or the Chicago Health and Aging Project (6 years follow-up) [21]. However, other studies about this topic show opposite results [22,23]. In a recent study in elderly Austrians [23], not relationship was found between vitamin B12 levels and cognitive decline; nevertheless, authors noted that the average concentrations of serum cobalamin in their sample were within the reference range. This aspect is in line with our results, where older adults with normal vitamin B12 levels did not experiment any MMSE score variation; conversely, subjects with abnormal values of vitamin B12 (<148 pmol/L) showed a significative deterioration on MMSE score.

On the other hand, several other studies reported a relationship between high tHcy and cognitive decline [24,25,26,27]. Differences in follow-up periods, cognitive test battery [27], or characteristics of the study population (e.g., free-living or institutionalized) [9,10] could explain some of the discrepancies among the studies.

According to our results, HDL-cholesterol was the only lipid biomarker showing an independent association with cognitive function. Low values of HDL-cholesterol correlated positively with poor MMSE scores. No significant differences were found for other lipids and lipoproteins. These results agreed with those obtained by other authors [28,29], who found that cognitive dysfunction is associated with a progressive decline in plasma HDL-cholesterol concentrations. This underscores the protective effects of increased plasma HDL-cholesterol and its role in maintaining superior cognition in longevity [30]. These positive associations demonstrate a significant role for lipid metabolism in preservation of cognitive function. Furthermore, implicating plasma HDL-cholesterol levels in initial cognitive decline, as well as the aforementioned decrease in sCbl at the end of life, may lead to the development of specific strategies to prevent this unfavorable condition.

### 4.3. Biomarkers and Physical Strength

We found no relationship between physical strength and biomarkers related to vitamin B12. These results are slightly in disagreement with a previous longitudinal study, which found no association with vitamin B12, but observed a significantly lower physical performance for women with high homocysteine levels [31].

In relation to parameters related to lipids, the present study showed that low levels of HDL-cholesterol were associated with poor physical strength. A Japanese study showed that changes in HDL-cholesterol were significantly and independently associated with changes in HGS in older people [32]. These results are similar to those obtained by other authors that in previous studies have demonstrated that low HDL-cholesterol levels are common in institutionalized or community-dwelling older adults with functional impairments [33,34,35]. Therefore, reduced HDL-cholesterol levels have been proposed as a general marker of “poor well-being” and reduced physical condition [36].

In another study carry out in older adult home residents, a combination of low HDL-cholesterol and low albumin was proposed as a means to diagnose frailty and predicted a 2.5 to 4-fold increase in short term mortality [37]. Similar results were found in our study in which low albumin and HDL-cholesterol levels were associated with low levels of muscle.

On the other hand, LDL-cholesterol concentrations were associated with better leg strength, high albumin concentrations with better strength parameters, and ApoA values with arm and leg strength testing results.

These results confirm their importance as health markers in the older adult. Our study has also shown an interesting fact. If we look at body parts separately and we apply the covariate model analysis, only high HDL-cholesterol values had a significant positive effect on HGS. This would mean that the association between biomarkers related to lipids and physical strength depends on the type of body parts analyzed.

### 4.4. Biomarkers, Cognitive Status, and Physical Strength

As reported previously [15], the present study showed an association between physical functions and cognitive status, which was maintained over time. In fact, several cross-sectional and longitudinal studies have sought relationships between HGS and cognitive status, finding that low levels of this type of strength are related to states of dementia [38,39,40,41,42].

Regarding biomarkers related to vitamin B12 status, no parameters were coincident between physical strength tests and cognitive status. Triglycerides were associated with the MMSE score and HGS; total-cholesterol was associated with HGS and, also showed a probability of 0.051 and 0.064 with MMSE and leg strength, respectively. LDL-cholesterol was associated with MMSE, HGS, and arm strength and showed a probability of 0.056 for leg strength. HDL-cholesterol was associated with MMSE and arm strength and with a probability of 0.058 for leg strength. ApoA was significantly associated with MMSE and leg and arm strength. Lastly, ApoB was only significantly associated with leg strength (*p* < 0.05). If we exclude HDL-cholesterol with HGS and ApoB, it can be said that all these parameters showed significant associations or trends in significance between them.

Albumin was the only biomarker that shows a positive correlation with MMSE and all physical strength tests. This is in line with previous studies that found that low albumin levels, even within the normal range, have been independently associated with worsening of muscle strength [43] and cognitive function [44]. Meanwhile, ApoB and ApoA are important predictors of cardiovascular risk and some studies have reported that they might outmatch the measurement of standard lipid parameters [45].

### 4.5. Strengths and Limitations

Longitudinal data on older people are still scarce. In this sense, this is the first longitudinal study analyzing the association of biomarkers related to vitamin B12 and lipid metabolism with physical strength and cognitive status in an older adult institutionalized population. In addition, the tests used to assess each domain are well established and recognized. However, the present study has several limitations that must be considered, such as the small sample size and the short follow-up period. Therefore, further studies should be conducted using a longer follow-up period in order to assess the utility of these biomarkers as predictive parameters of cognitive or physical strength declines. On the other hand, exclusion of patients who have started supplementation of B vitamins could have vitiated the results of the correlations by eliminating individuals with lower values. Moreover, although MMSE is a widespread and validated screening tool for dementia, a comprehensive neuropsychological battery would be necessary to capture all detailed aspects of cognitive function. Finally, it is important to highlight the elevated number of drugs taken by the participants in our study. In a previous work using the same population, we reported that daily medication intake was five drugs per participant, and some associations between drugs and blood parameters were observed [10].

## 5. Conclusions

Serum cobalamin, serum folate, HDL-cholesterol, and triglyceride levels show significant differences in the average values throughout a year and, except for folate, were associated with poor cognitive function.

Regarding physical strength, low values of HDL-cholesterol were associated with low HGS. Low albumin concentrations were observed in cases of poor leg strength, and low albumin and apolipoprotein A1 values were associated with low arm strength, confirming both serum albumin and strength as important health markers in the older adult. We found no relationship between physical strength and biomarkers related to vitamin B12.

The associations of low and high HGS levels with low and high HDL-cholesterol values, as well as the significance of negative correlations of LDL-cholesterol and triglycerides with HGS and arm strength, and the significance with the cognitive results, point to the convenience of arm and leg measurement, and especially HGS assessment, a very simple test, as an important health marker.

## Figures and Tables

**Table 1:** Baseline characteristics of the study population (*n* = 60) ^1^.

Gender	Overall
Men	19
Women	41
Age, years	
Men	80.6 (9.9)
Women	85.1 (5.5)
Weight, Kg	
Men	70.0 (11.0)
Women	59.3 (5.2)
BMI, kg/m^2^	
Men	27.9 (4.1)
Women	28.3 (5.2)
Abbreviations: BMI = body mass index	

^1^ Values are means (SD) or *n*.

**Table 2:** Pearson’s correlation coefficients (*p*) between biomarkers; cognitive and functional tests.

Biomarkers	MMSE Score	Handgrip Strength (kg)	Leg Strength (rep)	Arm Strength (rep)
Serum cobalamine, pmol/L	0.219 **	0.093	−0.065	0.078
Holotranscobalamin	−0.070	0.039	0.060	0.023
Serum folate, nmol/L	0.028	−0.048	−0.001	−0.001
RBC folate, nmol/L	−0.012	−0.171 *	−0.084	−0.101
Homocysteine, µmol/L	−0.077	−0.068	−0.042	−0.058
Creatinine, µmol/L	−0.046	0.085	−0.024	0.053
Triglycerides, mg/dL	−0.203 **	−0.139 *	0.067	−0.118
Total cholesterol, mg/dL	−0.132	−0.172 *	0.134	−0.054
LDL-cholesterol, mg/dL	−0.153 *	−0.162 *	0.138	−0.153 *
HDL-cholesterol, mg/dL	0.182 *	0.006	0.137	0.208 *
Glucose, mg/dL	−0.105	−0.021	−0.041	−0.016
Apolipoprotein A1, mg/dL	0.178 *	0.068	0.243 **	0.325 **
Apolipoprotein B, mg/dL	−0.091	−0.111	0.154 *	−0.072
Lipoprotein A, mg/dL	0.107	−0.126	0.018	−0.066
Albumin, g/dL	0.277 **	0.180 *	0.338 **	0.286 *

* *p* < 0.05; ** *p* < 0.001.

**Table 3:** MMSE score and functional tests results adjusted by biomarkers as covariate variables. Only significant effects (*p* < 0.05) are shown (linear mixed model).

Covariates	MMSE Score		Handgrip Strength (kg)		Leg Strength (kg)		Arm Strength (kg)	
	Param.	*p*	Param.	*p*	Param.	*p*	Param.	*p*
Serum cobalamin, pmol/L	0.009	0.011	-	-	-	-	-	-
HDL-cholesterol, mg/dL	0.151	<0.001	0.127	<0.001	-	-	-	-
LDL-cholesterol, mg/dL	-	-	-	-	0.026	0.031	-	-
Apolipoprotein A, mg/dL	-	-	-	-	-	-	0.072	<0.001
Albumin, g/dL	-	-	-	-	3.906	<0.001	2.039	0.014

Abbreviations: MMSE = Mini-mental state examination; Param. = parameter estimate; *p* = parameter significance.

## Supplementary Materials

The following are available online at https://www.mdpi.com/1660-4601/17/19/7303/s1. Table S1: Biomarkers and functional and cognitive tests evolution along a year.
