# Supplementary material for: Health Risk, Functional Markers and Cognitive Status in Institutionalized Older Adults: A Longitudinal Study
Source: Int J Environ Res Public Health. 2020 Oct 7;17(19):7303. doi: 10.3390/ijerph17197303 (PMC7579099; doi:10.3390/ijerph17197303)
Supplement: Supplementary file 1 [file ijerph-17-07303-s001.pdf]

Supplementary Material Table S1. Biomarkers and functional and cognitive tests evolution along a year.

	Month 1		Month 5		Month 9		Month 13	
	Median (IQR)	% abnormal[1]	Median (IQR)	% abnormal[1]	Median (IQR)	% abnormal[1]	Median (IQR)	% abnormal[1]
Parameters related to B12 status								
Serum cobalamine, pmol/L	286 (231-349)	10	275 (223-347)	5	252 (203-325)	10	235 (205-358)	10
Serum folate, nmol/L	14.0 (11.0-16.6)	70	15.2 (11.8-21.5)	55	15.4 (12.2-21.5)	53	15.7 (11.8-18.9)	50
RBC folate, nmol/L	779 (619-1003)	3	852 (654-1102)	4	872.5 (631.0-1108.6)	8	855.2 (659.9-1094.6)	12
Holotranscobalamin, pmol/L	46.0 (30.9-57.3)	32	46.2 (35.6-65.5)	25	42.8 (34.3-56.6)	28	47.7 (35.2-60.1)	25
Homocysteine, µmol/L	17.1 (13.9-21.5)	63	16.9 (14.7-23.1)	70	17.2 (14.1-25.6)	60	17.0 (14.0-24.2)	68
Parameters related to lipids								
Total cholesterol, mg/dL	189.0 (168.0-203.3)	30	194.5 (177.0-213.5)	43	197.0 (174.5-216.5)	46	194.0 (169.0-211.0)	38
HDL-cholesterol, mg/dL	49.5 (41.0-58.0)	13	49.0 (40.7-57.7)	10	46.5 (36.0-53.0)	22	47.0 (36.8-53.3)	22
LDL-cholesterol, mg/dL	116.1 (97.7-137.5)	33	122.3 (106.6-142.3)	43	126.0 (104.3-141.4)	46	121.0 (102.0-141.0)	38
Triglycerides, mg/dL	92.0 (68.5-121.0)	10	98.0 (75.7-128.2)	10	110.0 (87.5-138.0)	20	107.0 (83.8-131.0)	20
Apolipoprotein A1, mg/dL	132.5 (106.8-153.3)	17 / 0 [2]	148.5 (129.2-165.2)	3 / 5 [2]	143.0 (123.5-168.0)	7 / 3 [2]	135.0 (113.0-157.0)	12 / 3 [2]
Apolipoprotein B, mg/dL	87.5 (72.7-94.0)	3 / 0 [2]	91.5 (77.7-104.0)	0 / 0 [2]	96.0 (77.5-107.0)	3 / 2 [2]	87.5 (76.0-98.5)	2 / 0 [2]
Lipoprotein A, mg/dL	30.7 (17.9-75.3)	52	36.1 (19.1-75.6)	58	38.1 (20.3-57.7)	58	35.4 (19.1-78.8)	53
Other parameters								
Glucose, mg/dL	96.5 (91.0-108.3)	38	102.0 (91.0-108.5)	53	96.5 (95.6-105.0)	37	99.0 (93.0-108.0)	43
Albumin, g/dL	4.0 (3.7-4.3)	2 / 0 [2]	3.8 (3.7-4.4)	0 / 0 [2]	4.1 (3.8-4.4)	7 / 0 [2]	3.6 (3.4-4.0)	20 / 0 [2]
Creatinine, µmol/L	70.4 (61.6-96.8)	10	70.4 (61.6-96.8)	10	70.4 (52.8-88.0)	8	70.4 (61.6-99.0)	15
Functional tests								
Handgrip, Kg	10.6 (7.5-18.6)	-	11.6 (7.7-19.3)	-	10.7 (8.1-18.6)	-	11.9 (8.1-17.8)	-
Arm strength, rep	10.0 (5-13)	-	10.5 (6-15)	-	11.5 (6-14)	-	9.5 (5-14)	-
Leg Strength, rep	5 (0-8)	-	6 (0-10)	-	3.5 (0-11)	-	0 (0-9)	-
Cognitive test								
MMSE, score	17.5 (11.0-23.0)	-	16.0 (11.0-24.5)	-	17.0 (11.0-23.0)	-	16.0 (18.0-24.0)	-

Abbreviations: IQR = interquartile range.

[1] Abnormal values criteria: serum cobalamin <148 pmol/L; serum folate <15.8 nmol/L; RBC folate <362.4 nmol/L; Holotranscobalamin <35 pmol/L; homocysteine >15 µmol/L; total cholesterol >200 mg/dL; HDL-cholesterol <35 mg/dL; LDL-cholesterol >130 mg/dL; triglycerides >150 mg/dL; Apolipoprotein A1 out of 101-198 mg/dL range; Apolipoprotein B out of 52-163 mg/dL range; lipoprotein A >30 mg/dL; glucose >100 mg/dL; Albumin out of 3.3-5.0 g/dL range.

[2] Below / above normal range.

[3] Mean differences assessed using a mixed model with MMSE score and physical performance results as dependent variables.
